# Is HIV/AIDS a consequence or divine judgment? Implications for faith-based social services. A Nigerian faith-based university's study

**DOI:** 10.1080/17290376.2014.910134

**Published:** 2014-05-12

**Authors:** Israel B. Olaore, Augusta Y. Olaore

**Affiliations:** ^a^ PhD, are affiliated to Religious Studies, Department and School of Public and Allied Health, Social Work Program, Babcock University, Ilishan-Remo, Ogun State, Nigeria

**Keywords:** HIV/AIDS, Christian, university, consequence, judgment, VIH/SIDA, Chrétienne, université, conséquence, jugement

## Abstract

A contemporary reading of Romans 1:27 was disguised as a saying by Paul Benjamin, AD 58 and administered to 275 randomly selected members of a private Christian university community in south western Nigeria in West Africa. Participants were asked to respond to a two-item questionnaire on their perception of the cause of HIV/AIDS either as a judgment from God or consequence of individual lifestyle choices. The apparent consensus drifted in the direction of God as the culprit handing down his judgment to perpetrators of evil who engage in the homosexual lifestyle. The goal of this paper was to examine the implications of a judgmental stance on addressing the psychosocial needs of Persons Living with HIV/AIDS in religious environments. It also explores how service providers in faith-based environments can work around the Judgment versus Consequence tussle in providing non-discriminatory services to persons diagnosed with HIV/AIDS.

## Résumé

Une interprétation moderne de Romains 1:27 a été déguisée en adage par Paul Benjamin (58 Apr. J.-C.) et administrée de façon aléatoire à 275 membres de la communauté d'une université chrétienne privée au sud-ouest du Nigéria, en Afrique de l'Ouest. Il était demandé aux participants de répondre à un questionnaire à deux points concernant leur perception sur la cause du VIH/SIDA, qui est soit un jugement de Dieu, ou une conséquence des choix du mode de vie d'un individu. Le consensus s'est clairement penché du côté de Dieu, accusé d'exécuter son jugement envers les auteurs du mal qui se livrent à un style de vie homosexuel. L'objectif de cet article est d'examiner les implications d'une position critique en répondant aux besoins psychologiques des personnes porteuses du VIH/SIDA en milieux religieux. Il explore aussi comment les prestataires de services dans des environnements d'organisations religieuses peuvent contourner la dispute entre Jugement et Conséquence en pourvoyant des services non discriminatoires aux individus diagnostiqués avec le VIH/SIDA.

## Introduction

The Global Health Council (2011) estimates that over the last 27 years, nearly 25million people have died from HIV/AIDS. According to Avert-International HIV&AIDS Charity (2011), an estimated 23.5 million people were living with HIV in sub-Saharan Africa at the end of 2011, including 2.3 million children. Ninety percent of the 16.6 million children orphaned by AIDS live in sub-Saharan Africa.^1^ About 3.3 million people live with HIV in Nigeria, the most populous nation in sub-Saharan Africa, with a 3.6% adult HIV prevalence rate.^2^ The population of Nigeria is estimated to be about 162.5 million persons in 2011.^3^ According to the CIA-World Facts Book (2013), Nigeria is a multi-ethnic, religiously pluralistic nation with three main religious groups; Christianity – 40%, Islam – 50% and African Traditional Religion – 10%.^4^

## Literature review

UNAIDS, in a document ‘Religion and HIV’ in collaboration with the Ecumenical Alliance (e-alliance 2010), affirm that 70 percentage of the people in the world claim to belong to a faith community. In *UNAIDS:* ‘AIDS in Africa: Three scenarios to 2025’ (2005), the second of five critical and uncertain forces driving AIDS in Africa, expressed as ‘The Evolution of beliefs, values, and meanings’ focuses on traditional or religious systems and

individual beliefs about personal identity and morality, and about sexuality, illness, life, death and cosmology.^5^ The Ecumenical Advocacy Alliance Summit of High Level Religious Leaders in Response to HIV held in Den Dolder, The Netherlands, 22–23 March 2010 (e-alliance, 2010) affirmed with humility the ‘harm people had suffered in the name of religion’ with a pledge to commit themselves to strengthened efforts to respond to HIV.

Cooke (2005) in a report on Nigeria for the US Center for Strategic and International Studies highlighted the positive role of religion in the war on HIV/AIDS. Hence, the role of religion in the current war against the HIV/AIDS epidemic is one that needs to be fought on all fronts if victory is to be assured. UNAIDS (2011) estimates that faith-based organizations (FBOs) provide between 30% and 70% of all health care in Africa.^6^ This being the case, it is necessary to correct the impressions of people in FBOs about HIV/AIDS, people living with HIV (PLWHIV) and what the Bible says about HIV/AIDS. Surprisingly, few studies examine the impact of religion on AIDS-preventive behavior in West Africa even though religious beliefs and norms may be salient to AIDS prevention (Takyi 2003).

Letamo (2003) suggests that stigma and the resulting discriminatory attitudes create an environment that fuels the spread of HIV. Stigmatizing, non-acceptance and intolerance toward PLWHIV has been implicated as an obstacle in prevention programs and lack of eagerness in the education of unreached at-risk groups (Brooks, Etzel, Hinojos, Henry & Perez 2005; Chesney & Smith 1999; Parker & Aggleton 2003; Peretti-Watel, Spire, Obadia, Moatti, the VESPA Group 2007). This presents a strong case for seeking ways of measuring the levels of HIV/AIDS-related awareness and stigma ratio in a society so as to create the right avenues for dissipating the atmosphere of the erroneous beliefs before it serves as a negative factor in programs targeted at young people to reduce the spread of HIV/AIDS. Chiao, Mishra and Sambisa (2009) in a study on Kenya found that

accepting attitudes toward PLHIV are significantly associated with age, education, AIDS knowledge, perceived risk of getting infected with HIV, and knowing someone with HIV or someone who had died of AIDS, even after adjusting for other individual and community level variables. (749)

It has been observed that religious doctrines and moral positions by religious leadership have helped create and support perceptions that those infected have sinned and deserve their punishment, thus increasing the stigma associated with HIV/AIDS (Singh, 2001). It was also observed that while stigma can prevent people from seeking help, stereotypical beliefs about AIDS could also encourage complacency and reluctance to provide services (Takyi 2003). Women suspected of living with HIV/AIDS are considered promiscuous or as having been sex workers and thus live with the social dogma of deserving HIV infection (Wingood, Diclemente, Mikhail, McCree, Davies, Hardin, *et al*., 2007) thus undeserving of services. Herek, Capitanio, and Widaman (2002) stated that stigmatization and negative feelings toward people living with HIV/AIDS is associated with discomfort, blaming people with AIDS and misapprehensions about routes of HIV transmission.

## Motivation for study

At a recent conference of the Nigerian Association for Biblical Studies held July 2011, at Nassarawa State University, Keffi, Nassarawa State, Nigeria, with the theme ‘Contagious Diseases and Biblical Recipe’, two topics were listed along with a host of others that peaked the interest of the authors in deciding to write on the title of this paper. The topics were, *‘*Sexual vices as factors for the spread of sexually related diseases: A study of Romans 1:27 and 1 Cor. 6:9’ and ‘HIV/AIDS as Divine punishment for sin’.

‘And the men, instead of having normal sexual relationships with women, burned with lust for each other. Men did shameful things with other men and, as a result, suffered within themselves the penalty they so richly deserved’ (Romans 1:27 New Living Translation).^7^

Don't you know that those who do wrong will have no share in the Kingdom of God? Don't fool yourselves. Those who indulge in sexual sin, who are idol worshipers, adulterers, male prostitutes, homosexuals, thieves, greedy people, drunkards, abusers, and swindlers – none of these will have a share in the Kingdom of God. (1 Corinthians 6:9, 10 New Living Translation)^8^

The meanings of these biblical passages attributable to Apostle Paul the writer of Romans and 1 Corinthians may be taken for granted by Christians, but on a closer examination may evoke negative reactions about PLWHIV. This study examined the belief about AIDS as reflected in responses to Romans 1:27 and its impact on attitudes and service delivery in a faith-based community. The study will not be making reference to the 1 Corinthians text of the bible as a point of reference in the questionnaire administered to respondents in the study. The biblical passage given to respondents will be restricted to Romans 1:27 to reduce the incidence of confusion on the part of the respondents.

Given the generally increasing awareness that the virus does not discriminate by age, race, gender, ethnicity, sexual orientation or socioeconomic status, it is also a growing fact that certain groups are at particular risk of HIV, including men who have sex with men, injecting drug users and commercial sex workers (Global Health Council 2011). How then does a Christian community respond to the question of HIV/AIDS, as a consequence of personal lifestyle choices or a judgment of God on people with homosexual lifestyle? The challenge that this poses on a Christian institution or FBO is that there is a faith dilemma that complicates the fight against HIV/AIDS. It is therefore imperative to understand the cause of the stigma and to seek effective ways to tackle it. Greeley (1991) posited that there is a higher tendency to blame among people with high religiosity. Therefore, this study was carried out at Babcock University in Nigeria in the year 2011.

Babcock University Nigeria is a private Christian University owned and operated by the Seventh-day Adventist Church in West Africa. Babcock University is a multi-campus institution of higher learning with an undergraduate population of 8000 students and a post-graduate population of 550. There are over 300 persons that make up the teaching faculty, while the administrative staff is in excess of 1200 persons. The university serves the whole of Nigeria and sub-Saharan Africa. Of the 128 universities in Nigeria, 50 are private universities with Babcock University being one of the first to be chartered in 1999.^9^ Christian proprietors sponsor 21 of the private universities. The criteria for selecting the site included the need for it to be a Christian higher education institution of long standing since inception. Other universities within the criteria include Madonna University in Ananbra state, which was founded in 1999, and Covenant University in Otta, Ogun state, which was founded in 2002. Benson Idahosa University in Edo state and Bowen University in Osun state founded in 2002 and 2001, respectively, also fit within the selection criteria.^10^ Babcock University was chosen for its fit with the criteria and proximity to the investigators.

## Methodology

This is a qualitative case study design. According to Tashakkori and Teddlie (1998), this is an explorative case study. Site selection was made by the process of purposeful sampling (Creswell, 1998). The use of data display through matrixes, charts and networks has been suggested by Siedman (1991) and Miles and Huberman (1994).

The objective of the study in Nigeria was to measure the accepting attitudes toward PLHIV levels among students and staff at a private Christian university to determine if there was a positive correlation between religiosity and positive attitudes toward PLWHIV. A similar study by the team of Paruk, Mohamed, Patel and Ramgoon (2006) looking at South African Muslim students' attitudes to people with HIV/AIDS found that Muslim students displayed an unexpectedly tolerant attitude toward PLWHIV.

A qualitative study was conducted with 300 respondents made up of students and staff members at Babcock University, a private Christian University in Nigeria. A survey instrument referred to as the ‘Lifestyle and Consequences from a Religious Faith Perspective’ survey was designed to test the responses on – Roman 1:27. The one page two-question qualitative interview open-ended survey questions were preceded by a five-question socio-demographical survey questions that was administered by a research associate within a one-month period to requested volunteers in the five faculties of the university, covering two campuses. The survey sought the informed consent of the respondent while guaranteeing confidentiality and anonymity. A total of 275 of the 300 surveys were returned for processing. Of the 275, 79 were staff members while 197 were students.

## Distribution

Of the 300, 267 responding to the socio-demographical question requesting for gender information indicated 116 or 43% were male while 151 or 57% were female. Of 273 responding to the question on age range, 158 respondents or 58% were within the ages of 15–25 years, 86 or 32% were in the 26–40 age range. Twenty-one or 8% were in the 41–50 age range. Five or 2% were in the 51–60 range, while 3 or 1% were in the 60 and above age range. The age distribution demonstrates that the majority of the respondents (58%) were students. Of the students 11% were in high school, 71% were in undergraduate programs, 18% were in the post-graduate program. The majority of the respondents identified themselves as Christian (98.1%) and were single (75.7%).

## Findings

To test the knowledge base of the respondents regarding the opinion if there is any relationship between homosexual lifestyles and HIV/AIDS the following question was asked:

‘In your opinion is there a connection between the homosexual and gay lifestyle and HIV/AIDS. [Human Immunodeficiency Virus (HIV) – Acquired Immune Deficiency Syndrome (AIDS).] Please explain.’

63% indicated yes and 22% indicated no while 15% stated, I do not know.

**Fig. 1.**
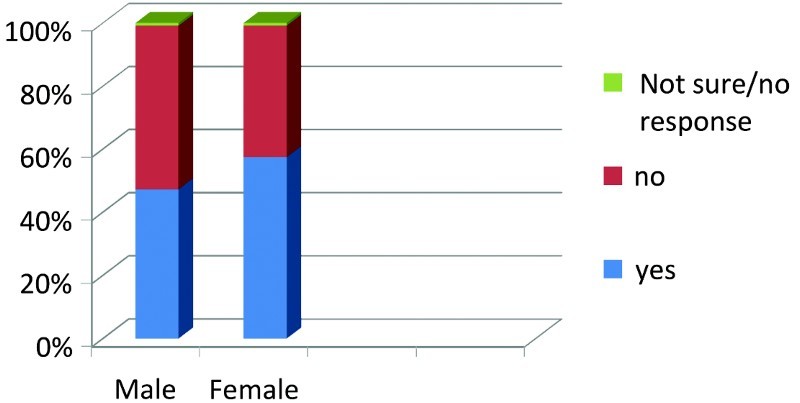
There is a connection between HIV/AIDS and Homosexual lifestyle.

Between genders, more female respondents stated that there is a connection between the homosexual lifestyle and HIV/AIDS as illustrated in Fig. 1.

**Fig. 2.**
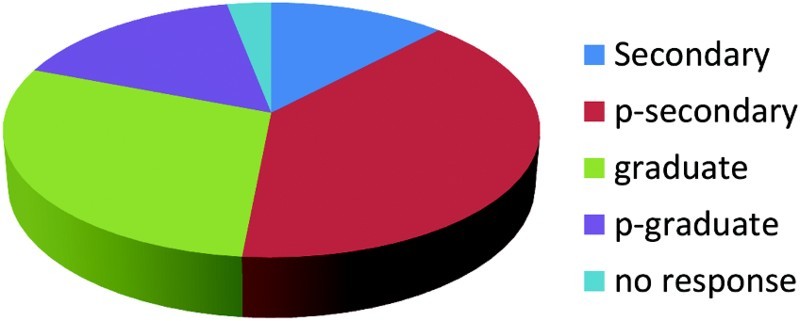
HIV/AIDS is God's punishment for gay/lesbian lifestyle.

In response to the question, ‘In your opinion, do you believe that HIV/AIDS is God's punishment for the participants of gay/lesbian lifestyle?’ most of the female respondents believed that HIV/AIDS is a punishment from God as shown in Fig. 2.

Thirty percent of the respondents who stated that HIV/AIDS was a punishment from God on persons involved with homosexual life style were students in post secondary institutions.

A female university student between ages 26 and 40 said ‘Yes, it's likely that bacterias can form into HIV after sexual intercourse.’

A male staff member also in the age range of 26–40 stated with a certainty of knowledge ‘Yes, because it's been recognized as a primary mode of transmission.’

A female Christian student within the age range of 15–25 confessed her limited knowledge by stating, ‘I don't think so. The virus started when a white lady slept with a Gorilla/Monkey.’

A male Christian student within the age range of 15–25 responded no to the question stating, ‘No, because AIDS is sexually transmitted between opposite sex.’

A female Christian student within the age range of 15–25 responded ‘No, because HIV/AIDS came to being by a man who slept with a dog. I feel it is transferred from man to woman not from man to man.’

The second question sought to elicit the opinion of the respondent on their understanding of a biblical statement disguised as a writing of one of the early Church fathers:

‘And the men, instead of having normal sexual relationships with women, burned with lust for each other. Men did shameful things with other men and, as a result, suffered within themselves the penalty they so richly deserved’ (Romans 1:27 New Living Translation).

**Fig. 3.**
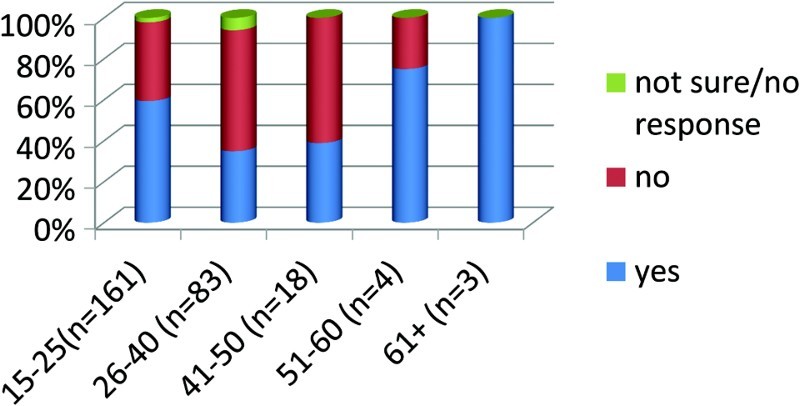
Do you believe that HIV/AIDS is God's punishment to participants of gay/lesbian lifestyle?

In your opinion do you believe from this statement that HIV/AIDS is God's punishment for the participants in the lifestyle? Please explain (Fig. 3).

Of 263 respondents, 128 or 49% responded that it was God's judgment, 118 or 45% did not think it was God's judgment and 17 or 6% claimed they did not know the answer.

A male Christian student stated that ‘HIV/AIDS is not God's punishment for the participant but it's punishment for disobeying God's word.’

Is there a connection between HIV/AIDS and homosexual lifestyle? Sample of respondents statements

- I do not think so. the virus started when a white lady slept with a Gorilla/Monkey.
- No, because it can only be transferred from male to female.
- Yes, because in order to satisfy their sexual urge, men go after animals and men.
- Yes, there's a connection because it is a sinful attitude.
- No, there's no relationship between homosexuals and gay lifestyle with HIV/AIDS even though the lifestyle is not healthy and also not godly.
- Yes, there is a connection, because originally, HIV was actually gotten from there, by sleeping with dogs, the same sex, etc.
- These are satanic ways of creating in people's mind bad image about our creator. God ordained man to woman relationship not man to man.

## Discussion

As Agadjanian (2001) has pointed out, church participation in Africa creates an environment for social exposure and interaction to new ideas, which could then influence AIDS prevention. These responses reflect the need for an extensive educational program in training university students about the various modes of infection and transmission as well as prevention strategies for avoiding contracting HIV through reckless behavior.

The study of Hekel *et al.* (2002) was confirmed in this study that erroneous belief about HIV/AIDS could fuel stigmatization. Some of the respondents stated that HIV/AIDS came as a result of a human having sexual intercourse with an animal. There is therefore an ongoing need to educate young people about the etiology of AIDS/HIV especially in post secondary institutions. Current epidemiological studies have established three modes of transmission of HIV/AIDS, which are sexual intercourse with an infected person; second, through exposure to contaminated blood, blood products or transplanted organs and tissues; and third, from an infected mother to her fetus or infant before, during or shortly after birth (Amosu, Degun, Makinde, Thomas & Babalola 2011).

It was also observed that the view of AIDS/HIV as a punishment from God was largely held by women in this study. It is therefore recommended that faith-based psycho-educational intervention target women. Studies have shown that women are more likely than men to be found in Christian religious settings and be involved in providing social services more than men (Harlow 2004; Loewenthal, MacLeod & Cinnirella 2002; Parsons, Cruise, Davenport & Jones 2006). Perceptions that are rooted in religious beliefs may lead to the demonstration of discriminatory behaviors among healthcare providers as observed by Amosu *et al.* (2011). This study, therefore, agrees that it is pertinent that healthcare providers have access to accurate and updated information as to the modes of transmission, counseling and guidelines for safe practice. This psycho-educational intervention should target women in religious settings as either current or potential caregivers. This may reduce the tendency for stigmatization and belief that PLWHA are deserving of punishment and not treatment or services.

Christian faith-based institutions may choose to extend their teaching to include the attitude of Jesus the leader of Christianity to vulnerable and marginalized persons in the society. The New Testament books of Matthew through to Luke reveal examples of him reaching out and deliberately touching a leper, Matthew 8:2–3. In another instance he was accused of hanging out with prostitutes and social outcasts, Luke 15:1–3. He was also accused of allowing a prostitute to touch him, Luke 7:37–50. In these instances we are made aware that Jesus did not discriminate on the basis of social standing and perceived sinfulness of persons who were otherwise viewed as social outcasts.

## Recommendations for FB social services

1. That psycho-educational intervention on HIV/AIDS be carried out in churches and other religious settings which will inform persons, especially women, who may be potential or current caregivers or service providers for PLWAH. These may also prepare a pool of well-informed personnel for Faith-Based AIDS/HIV initiatives.
2. Targeting youth and young adults. As this study has revealed youth and young adults in Nigeria have erroneous perceptions about the etiology of AIDS/HIV. Access to accurate information among this population may reduce risky sexual behaviors as well as reduce stigmatization of PLWAH. Faith-based higher education institutions are encouraged to be in the forefront of educating young people about AIDS/HIV.
3. Faith-based initiatives to encourage disclosure. The openness of religious leaders to talking about issues and needs of PLWAH as suggested in this study may create an environment where PLWAH may feel more comfortable to disclose and seek for services in faith-based settings more promptly.
4. It is recommended that religious leaders assist their congregants to extend existing traditional beliefs and mandates for caring to address psychosocial needs of PLWAH rather than they being treated as outcasts deserving of God's judgment or punishment.
5. It is equally worthy of mention that Jesus Christ in dealing with vulnerable persons in his day on two occasions (with the paralyzed man by the pool of Bethesda in John 5:1–8 who had been sick for 38 years and the woman caught in adultery in John 8:1–12 and brought to Jesus for judgment) gave them the admonition to ‘go and sin no more’. This admonition was a two-edged statement of empowerment to live a sin-free as well as condemnation-free life. It was also an indication that they remove themselves from situations and circumstances that precipitated and predicated infection or re-infection of disease, in this case with HIV. It is therefore suggested that part of the educational process for the target population be focused on sexual abstinence training and faithfulness to single sexual partner activity.

## Limitations of study

One of the limitations of this study is that it was carried out in a Christian private university. The findings may not be generalizable to other religious settings such as Islam or traditional religion. Also it was restricted to the university community. A follow-up research may be carried out in church-based settings. The small sample size of 300 questionnaires may also be viewed as a limitation. Finally, the instrument utilized was a two-point questionnaire, which may have been simple for respondents to understand. The format of the questionnaire may not have allowed for the capture of more details about behavior among respondents.

## Conclusion

This study was carried out in a faith-based University in Nigeria to explore the beliefs and perceptions about AIDS/HIV in response to a contemporary reading of Roman 1: 27. A majority of the respondents believed that AIDS/HIV was a punishment from God and erroneous beliefs about causality and modes of transmission were expressed. The study made recommendations for FBOs to provide a forum for accurate education and socialization of congregants to be open to providing services to PLWHA. Religious leaders were encouraged to extend the Christian tenets of caring just as Jesus himself demonstrated to include services to PLWAH.

## Notes

1. Avert.org accessed 30 October 2013 at http://www.avert.org/worldwide-hiv-aids-statistics.htm.

2. prb.org/Publication/Datasheet/2009/2009wpds.aspx. Accessed 7 November 2011.

3. tradingeconomics.com/Nigeria/population, accessed 30 October 2013, http://www.tradingeconomics.com/nigeria/population-total-wb-data.html.

4. CIA-World Facts Book (2013), accessed 30 October 2013, https://www.cia.gov/library/publications/the-world-factbook/geos/ni.html.

5. UNAIDS/04–52E (English original, January 2005, p. 14) Joint United Nations Programme on HIV/AIDS (UNAIDS) 2005

6. Religion and HIV, UNAIDS (2011) http://www.e-alliance.ch/typo3conf/ext/naw_securedl/secure.php?u=0&file=fileadmin/user_upload/docs/UNAIDS_Religion_and_HIV.pdf accessed 17 November 2011.

7. Romans 1:27 New Living Translation – from Bible Works 6 (2006b).

8. 1 Corinthians 6:9 New Living Translation – from Bible Works 6 (2006a).

9. 
http://www.nuc.edu.ng/pages/universities.asp?ty=3&order=inst_name&page=2.

10. 
http://jeteraps.scholarlinkresearch.org/articles/Private%20Universities%20in%20Nigeria.pdf.
